# Comparative responsiveness of the PROMIS-10 Global Health and EQ-5D questionnaires in patients undergoing total knee arthroplasty

**DOI:** 10.1302/0301-620X.101B7.BJJ-2018-1543.R1

**Published:** 2019-06-30

**Authors:** J. Shim, D. F. Hamilton

**Affiliations:** 1 Epidemiology Group, School of Medicine, Medical Sciences and Nutrition, University of Aberdeen, Aberdeen, UK; 2 Aberdeen Centre for Arthritis and Musculoskeletal Health, University of Aberdeen, Aberdeen, UK.; 3 Department of Orthopaedics and Trauma, School of Clinical Sciences, University of Edinburgh, Edinburgh, UK.

**Keywords:** Knee arthroplasty, Health-related quality of life, Outcome measures, PROMIS-10 Global Health, EQ-5D, Responsiveness

## Abstract

**Aims:**

Responsiveness to clinically important change is a key feature of any outcome measure. Throughout Europe, health-related quality of life following total knee arthroplasty (TKA) is routinely measured with EuroQol five-dimension (EQ-5D) questionnaires. The Patient-Reported Outcomes Measurement Information System 10-Question Short-Form (PROMIS-10 Global Health) score is a new general heath outcome tool which is thought to offer greater responsiveness. Our aim was to compare these two tools.

**Patients and Methods:**

We accessed data from a prospective multicentre cohort study in the United Kingdom, which evaluated outcomes following TKA. The median age of the 721 patients was 69.0 years (interquartile range, 63.3 to 74.6). There was an even division of sex, and approximately half were educated to secondary school level. The preoperative EQ-5D, PROMIS-10, and Oxford Knee Scores (OKS) were available and at three, six, and 12 months postoperatively. Internal responsiveness was assessed by standardized response mean (SRM) and effect size (Cohen’s *d*). External responsiveness was assessed by correlating change scores of the EQ-5D and PROMIS-10, with the minimal clinically important difference (MCID) of the OKS. Receiver operating characteristic (ROC) curves were used to assess the ability of change scores to discriminate between improved and non-improved patients.

**Results:**

All measures showed significant changes between the preoperative score and the various postoperative times (p < 0.001). Most improvement occurred during the first three months, with small but significant changes between three and six months, and no further change between six and 12 months postoperatively. SRM scores for EQ-5D, PROMIS-10, and OKS were large (> 0.8). ROC curves showed that both EQ-5D and PROMIS-10 were able to discriminate between patients who achieved the OKS MCID and those who did not (area under the curve (AUC) of 0.7 to 0.82).

**Conclusion:**

The PROMIS-10 physical health tool showed greater responsiveness to change than the EQ-5D, most probably due to the additional questions on physical health parameters that are more susceptible to modification following TKA. The EQ-5D was, however, shown to be sensitive to clinically meaningful change following TKA, and provides the additional ability to calculate health economic utility scores. It is likely, therefore, that EQ-5D will continue to be the global health metric of choice in the United Kingdom.

Cite this article: *Bone Joint J* 2019;101-B:832–837.

Osteoarthritis (OA) of the knee is a common and disabling condition that may ultimately require surgical intervention. More than 100 000 total knee arthroplasties (TKA) are performed each year in the United Kingdom alone.^1^ This number is expected to increase year on year.^2,3^ Although TKA is generally effective at reducing pain and improving function in patients with end-stage OA, some patients report dissatisfaction with the outcome,^4,5^ with persistent physical impairment^6^ and limitations of activity.^7-10^ It is thus vitally important to use appropriate metrics when reporting changes in symptoms and outcome prior to, and following, TKA. Patient-reported outcome measures (PROMs) are increasingly used to assess outcome. These questionnaires evaluate aspects of health, function, and quality of life from the perspective of the patient.^11^ General health or health-related quality-of-life (HRQoL) PROMs are typically used in combination with joint-specific or condition-specific scores in national data sets to generate the broadest picture of function, and to allow comparison with other conditions and forms of treatment.

In the United Kingdom, the current metric of choice for evaluating HRQoL is the EuroQol five-dimension score (EQ-5D). This is commonly used as it allows the calculation of quality-adjusted life years that are central to health-economic evaluation. The most used version is the EQ-5D-3L. Although its reliability and reproducibility have been well validated,^12-14^ its responsiveness in patients who have undergone arthroplasty is somewhat limited.

The Patient-Reported Outcomes Measurement Information System 10 (PROMIS-10) Global Health survey is a ten-item questionnaire that assesses generic HRQoL compared with normal values for the general population.^15^ It was developed by the United States National Institute of Health to evaluate HRQoL, and is contrasted against United States normative scores. It measures five domains: physical function, fatigue, pain, emotional distress, and social health on a five-point response matrix. The structure of the score should offer greater responsiveness to changes in general health.^16^

The international group, Outcome Measures in Rheumatology (OMERACT), which defines core outcome measurement sets in rheumatic diseases, recognized responsiveness to clinically important change as a key feature of any clinical outcome measure. Responsiveness is defined as the ability of an instrument to measure change over time.^17^ Comparative evaluation of responsiveness of the PROMIS-10 Global Health score and EQ-5D has not been conducted in patients undergoing TKA. The aim of this study, therefore, was to compare the responsiveness of these HRQoL metrics and to compare them with a joint-specific score, to determine responsiveness.

## Patients and Methods

Data from a prospective multicentre cohort study in the United Kingdom, investigating outcome following TKA (TRIO-POPULAR), were accessed. This study involved 721 patients undergoing primary TKA for OA from nine centres in the United Kingdom.^18^ This data set was chosen due to the nationally representative sample and extent of data assessment time points. Patients were evaluated preoperatively, and at three, six, and 12 months following surgery. The median age of the 721 patients was 69.0 years (interquartile range, 63.3 to 74.6). There was an even division of sex, and approximately half were educated to secondary school level (Table I). Ethical approval was granted by the office for Research Ethics Committees Northern Ireland (ORECNI) (13/NI/0101).

**Table I.** Baseline characteristics of the patients

Predictors	All patients	Patients lost to follow-up
Median age, yrs (IQR); n	69.0 (63.3 to 74.6); 721	69.1 (34.8 to 73.8); 17
Female sex, n (%)	379 (*52.6*)	15/17 (*88.2*)
**Education, n (%)**	**719**	**17**
Secondary school	356 (*49.5*)	11 (*64.7*)
Apprenticeship	81 (*11.3*)	1 (*5.9*)
Further education college	188 (*26.2*)	5 (*29.4*)
University degree	69 (*9.6*)	0 (*0*)
Further degree	25 (*3.5*)	0 (*0*)
**Marital status, n (%)**	**719**	**17**
Single	35 (*4.9*)	0 (*0*)
Married	485 (*67.5*)	11 (*64.7*)
Widowed	100 (*13.9*)	1 (*5.9*)
Divorced	67 (*9.3*)	4 (*23.5*)
Separated	8 (*1.1*)	0 (*0*)
Cohabiting	24 (*3.4*)	1 (*5.9*)
**Clinical factors**		
Median duration of knee pain, yrs, (IQR); n	5.0 (2.0 to 10.0); 699	5.5 (2.5 to 11.5); 16
Median baseline Oxford Knee Score, score (IQR); n	21.0 (15.0 to 26.0), 709	24.0 (16.0 to 28.0); 14
Median EQ-5D score, (IQR); n	0.42 (0.25 to 0.42); 699	0.42 (0.25 to 0.46); 14
Median EQ-5D VAS score, (IQR); n	75.0 (62.0 to 85.0); 691	70.0 (65.0 to 86.0); 14
Median PROMIS Global Physical Health, score (IQR); n	37.4 (34.9 to 39.8); 706	37.4 (34.9 to 39.8); 15
Median PROMIS Global Mental Health, score (IQR); n	45.8 (41.4 to 48.3); 712	43.5 (41.1 to 49.6); 16
**Comorbidities, n (%)**	**721**	
⩽ 1	175 (*24.3*)	10 (*58.8*)
2 to 3	421 (*58.4*)	6 (*35.3*)
≥ 4	125 (*17.3*)	1 (*5.9*)

IQR, interquartile range; EQ-5D, EuroQol five-dimensions; VAS, visual analogue scale; PROMIS, Patient-Reported Outcomes Measurement Information System

The EQ-5D-3L consists of a descriptive system with five dimensions: mobility, self-care, usual activities, pain/discomfort, and anxiety/depression, with a three-option response format, and the EQ-5D visual analogue scale (VAS). Each EQ-5D profile was converted to a single summary index based on the evaluation of health states in the United Kingdom. A score of 1.0 indicates best possible health, while negative values represent a health status worse than death. Separate to the EQ-5D profile, the EQ-5D VAS is a quantitative measure of the patients’ self-assessment of their health on a visual analogue scale (0 being worst, 100 being best).

The PROMIS-10 Global Health also measures five domains: physical function, fatigue, pain, emotional distress, and social health. Items are rated on a five-point scale. It includes physical and mental health component scores that can be transformed to *t *score distributions with a mean of 50 and standard deviation of 10. A higher score indicates better health.

Joint-specific measures are designed to capture the influence of interventions on the joint in question. They tend to be more responsive to the effect of interventions than generic scores. The Oxford Knee Score (OKS) is a commonly used PROM that has been validated to measure the impact of pain and functional disability in patients undergoing knee arthroplasty.^19^ The score consists of 12 items that evaluate pain and function, with five possible response options from 0 to 4. The summed total is reported (0 to 48), higher scores reflecting less pain and better function.

### Statistical analysis

We evaluated responsiveness by performing paired Student’s *t*-tests of the change in scores (postoperative score minus preoperative score). Percentage change was defined as the mean change scores divided by the baseline scores. Responsiveness was also assessed by the standardized response mean (SRM) and effect size using Cohen’s *d*.^20^ SRM was calculated by dividing the mean change score by the standard deviation of the change score, and effect size was calculated by dividing the mean change score by the standard deviation of baseline (preoperative) scores. An effect size of 0.2 is considered a small effect size, 0.5 is considered moderate, and > 0.8 is considered large.^20^ A bias-corrected bootstrap method with 2000 iterations was used to compare the differences in responsiveness estimates (SRM and effect size) between the measures.^21,22^ Bootstrapping is a resampling technique to draw numerous samples from the original sample with replacement.^23^ Bias-corrected 95% confidence intervals for these differences were obtained.^24^

We determined external responsiveness by correlating the six-month change scores of the EQ-5D and PROMIS-10 with the minimal clinically important difference (MCID) of the OKS. It is widely accepted that the postoperative OKS score plateaus after six months.^25^ The MCID is the minimal change in a score that is perceived by the patient to be beneficial,^26^ and is defined as more than five points for the OKS using the anchor method approach.^27^ Receiver operating characteristic (ROC) curves were used to assess the ability of change scores to discriminate between improved and non-improved patients, defined by the external criterion (dichotomized outcome of patients with an OKS MCID > 5). An area under the curve (AUC) value of 0.5 indicates a discriminatory value equivalent to chance.

Correlations between the six-month postoperative change scores of EQ-5D, PROMIS-10, and OKS were tested using Pearson’s R correlation. All statistical analysis was undertaken in STATA version 14.0 (StataCorp, College Station, Texas, 2015). Statistical significance was set at p < 0.05.

## Results

**Fig. 1:**
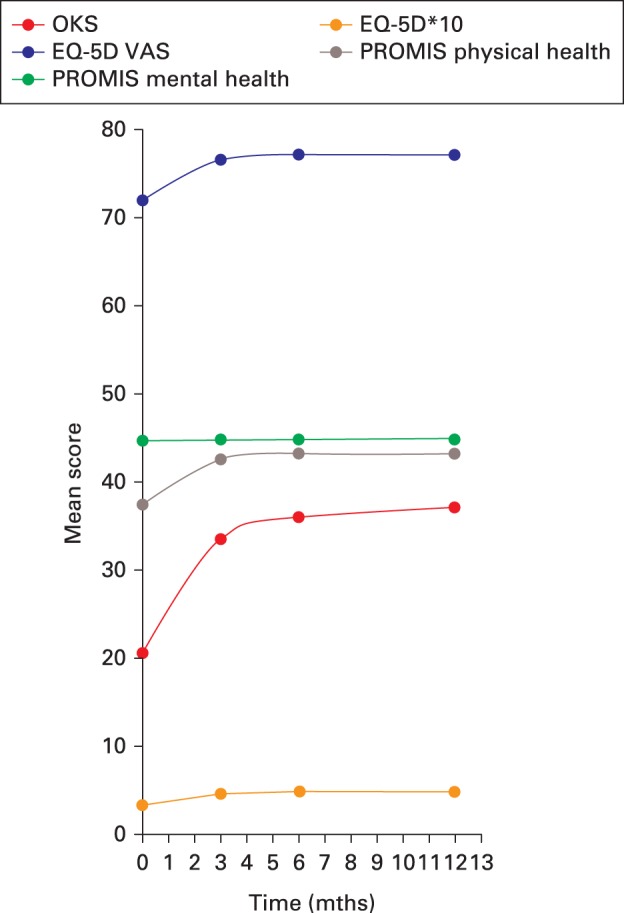
Chart showing the mean outcome score by time following total knee arthroplasty. OKS, Oxford Knee Score; EQ-5D, EuroQol five-dimensions; VAS, visual analogue scale; PROMIS, Patient-Reported Outcomes Measurement Information System.

Preoperatively, the median OKS score was 21.0, the median EQ5D quality of life score was 0.42, and the median physical health score was 37.4 (PROMIS Global Physical). The improved scores following surgery are shown in Figure 1. Most improvement in joint-specific function occurred in the early postoperative period and plateaued after six months. This change was captured to a lesser extent by the generic scores.

Patients who were lost to follow-up did not significantly differ in baseline age, sex, OKS, and health status (EQ-5D and PROMIS-10) (Table I), and no significant bias was assumed due to loss to follow-up.

### Internal responsiveness

The OKS, EQ-5D, EQ-5D VAS, and PROMIS-10 physical health component scores all showed significant changes between the preoperative score and postoperative times (p < 0.001; Table II). Most improvement occurred during the first three months in all patients, with small but significant changes between three and six months, and no further statistical change in any scores occurred between six and 12 months (Fig. 1 and Table II). Notably, the PROMIS-10 mental health component showed no change at any time compared with the preoperative values.

**Table II.** Mean change score for each measure between the preoperative value and different times postoperatively

PROMs	Mean 3 mths postoperative change score (95% CI); p-value^*^	Mean 6 mths postoperative change score (95% CI); p-value^*^	Mean 12 mths postoperative change score (95% CI); p-value^*^	Change in score between 3 and 6 mths (95% CI); p-value^*^	Change in score between 6 and 12 mths (95% CI); p-value^*^
OKS	12.68 (12.01 to 13.36); < 0.001	15.45 (14.79 to 16.12); < 0.001	16.27 (15.56 to 16.98); < 0.001	2.74 (2.32 to 3.16); < 0.001	0.81 (0.41 to 1.20); < 0.001
EQ-5D	0.12 (0.11 to 0.13); < 0.001	0.14 (0.13 to 0.16); < 0.001	0.15 (0.14 to 0.17); < 0.001	0.03 (0.02 to 0.03); < 0.001	0.01 (-0.003 to 0.02); 0.168
EQ-5D VAS	4.40 (2.99 to 5.82); < 0.001	5.28 (3.90 to 6.66); < 0.001	4.84 (3.39 to 6.29); < 0.001	1.03 (-0.08 to 2.14); 0.068	-0.97 (-2.13 to 0.19); 0.102
PROMIS Physical Health	4.97 (4.60 to 5.33); < 0.001	5.60 (5.25 to 5.95); < 0.001	5.57 (5.17 to 5.97); < 0.001	0.73 (0.44 to 1.03); < 0.001	-0.06 (-0.36 to 0.24); 0.677
PROMIS Mental Health	0.03 (-0.34 to 0.40); 0.875	0.18 (44.16 to 44.98); 0.349	0.11 (-0.29 to 0.52); 0.583	0.15 (-0.09 to 0.38); 0.215	-0.10 (-0.33 to 0.14); 0.421

* Paired-samples Student’s *t*-tests

PROMs, patient-reported outcome measures; CI, confidence interval; OKS, Oxford Knee Score; EQ-5D, EuroQol five-dimensions; VAS, visual analogue scale; PROMIS, Patient-Reported Outcomes Measurement Information System

The OKS showed significantly greater SRM and effect sizes compared with the other scales. In accordance with Cohen’s criteria, the SRM scores for OKS, EQ-5D, EQ-5D VAS, and PROMIS-10 physical were large (SRM > 0.8), indicating large changes in these measures over time, and they remained responsive at 12 months postoperatively. In contrast, the responsiveness of the VAS component of the EQ-5D and PROMIS-10 mental health was minimal to small (0.0 to 0.3), indicating little or no change over time (Table III).

**Table III.** Responsiveness estimates of effect size and standardized response mean (SRM) of the Oxford Knee Score (OKS), EuroQol 5 dimensions (EQ-5D), and Patient-Reported Outcomes Measurement Information System (PROMIS)-10

PROMs	Effect size 3 mths postoperative, Cohen *d*, (95% CI); p-value	Standardized response mean 3 mths postoperative, (95% CI); p-value	Effect size 6 mths postoperative; Cohen *d*, (95% CI); p-value	Standardized response mean 6 mths postoperative (95% CI); p-value	Effect size 12 mths postoperative, Cohen *d*, (95% CI); p-value	Standardized response mean 12 mths postoperative (95% CI); p-value
OKS	1.63 (1.51 to 1.75); < 0.05	1.44 (1.33 to 1.55); < 0.05	1.95 (1.84 to 2.08); < 0.05	1.73 (1.61 to 1.85); < 0.05	1.97 (1.82 to 2.12); < 0.05	1.78 (1.64 to 1.92); < 0.05
EQ-5D	0.80 (0.71 to 0.88); < 0.05	0.72 (0.64 to 0.80); < 0.05	0.92 (0.83 to 1.01); < 0.05	0.83 (0.74 to 0.92); < 0.05	0.98 (0.88 to 1.08); < 0.05	0.87 (0.77 to 0.96); < 0.05
EQ-5D VAS	0.26 (0.17 to 0.34); < 0.05	0.24 (0.16 to 0.32); < 0.05	0.30 (0.22 to 0.38); < 0.05	0.29 (0.21 to 0.37); < 0.05	0.27 (0.19 to 0.35); < 0.05	0.26 (0.18 to 0.34) < 0.05
PROMIS Physical Health	1.16 (1.06 to 1.27); < 0.05	1.06 (0.96 to 1.15); < 0.05	1.30 (1.19 to 1.40); < 0.05	1.20 (1.11 to 1.32); < 0.05	1.22 (1.11 to 1.34); < 0.05	1.09 (0.98 to 1.19); < 0.05
PROMIS Mental Health	0.01 (-0.07 to 0.08)^*^	0.01 (-0.07 to 0.08)^*^	0.04 (-0.03 to 0.12)^*^	0.04 (-0.03 to 0.12)^*^	0.02 (-0.06 to 0.10)^*^	0.02 (-0.06 to 0.10)^*^

* Not statistically significant (p > 0.05)

PROMs, patient-reported outcome measures; CI, confidence interval; VAS, visual analogue scale

### External responsiveness

**Fig. 2:**
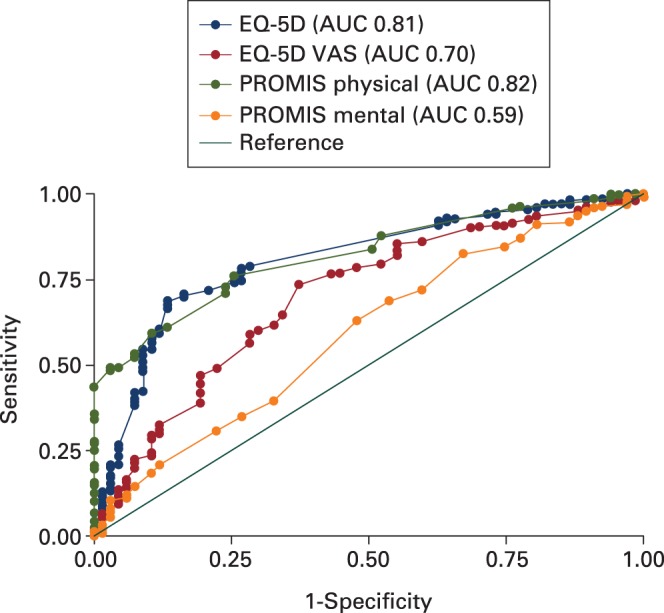
Receiver operating characteristic (ROC) curves for EuroQol five-dimensions (EQ-5D), EQ-5D visual analogue scale (VAS), and Patient-Reported Outcomes Measurement Information System (PROMIS) physical and mental health against the external criterion of a minimal clinically important difference (MCID) of a change in Oxford Knee Score (OKS) of greater than 5 points.

Positive correlations were observed for all measures with the OKS (p < 0.001) (Table IV). The OKS correlated most with the PROMIS-10 Physical Health and the EQ-5D measures. The change in scores correlated only weakly with the change in OKS score. ROC curves showed that EQ-5D, EQ-5D VAS, and PROMIS-10 physical health were all able to discriminate between patients who achieved the OKS score MCID (> 5) and those who did not (AUC 0.7 to 0.82). PROMIS-10 mental health showed poorer discriminatory ability (AUC 0.59; Fig. 2).

**Table IV.** Correlations with change in Oxford Knee Score (OKS) at six months; external responsiveness

PROMs 1	PROMs 2	n	Correlation (r)	95% CI	p-value
Change in OKS	Change in EQ-5D	649	0.51	0.45 to 0.57	< 0.001
	Change in EQ-5D VAS	647	0.30	0.24 to 0.37	< 0.001
	Change in PROMIS Physical Health	674	0.57	0.51 to 0.62	< 0.001
	Change in PROMIS Mental Health	648	0.14	0.06 to 0.22	< 0.001

PROMs, patient-reported outcome measures; CI, confidence interval; EQ-5D; EuroQol 5 dimensions; VAS, visual analogue scale; PROMIS, Patient-Reported Outcomes Measurement Information System

## Discussion

We confirmed good responsiveness of the PROMIS-10 Global Health score when used for the evaluation of patients undergoing TKA. As expected, the joint-specific tool (OKS) showed the greatest responsiveness to change following TKA. Both HRQoL measures were responsive to change following TKA. However, the PROMIS-10 physical health tool showed greater responsiveness than the EQ-5D, with the change in mean score, SRM, and effect size at all times, and the correlation to the joint-specific tool all greater in the PROMIS-10 physical health score compared with the EQ-5D. This difference is most likely to be due to the additional questions and focus on parameters of physical health, which are more susceptible to modification following surgery to the knee than the evaluation offered by the EQ-5D.

As expected, the mental health component of the PROMIS-10 tool showed no difference over time following TKA. Most change in score in all other measures occurred during the first three months after surgery. There were small but statistically significant further changes in PROMIS-10, EQ-5D, and OKS scores between three and six months, while only the OKS recorded change between six and 12 months.

Although other studies have evaluated the responsiveness of the OKS and EQ-5D in arthroplasty,^28,29^ this is the first to evaluate and compare the responsiveness of the PROMIS-10 Global Health questionnaire in patients undergoing TKA. This is also the first analysis to record the ability of the health-related quality of life tools to detect a clinically meaningful change in the OKS.

Typically, the assessment of responsiveness can provide an indication of whether a measure can detect a statistically significant change over time. Statistical significance does not, however, indicate whether this change is meaningful.^30^ Evaluating the comparative effect size and SRM of the health-related quality of life tools without an anchor of important change provides no information about the ability of the tools in question to measure change in the underlying construct.^31^ We assessed this external responsiveness by evaluating the ability of the health-related quality-of-life measures to affect the accepted clinically meaningful change of five points on the OKS. Although the PROMIS-10 physical health component score correlated most with the OKS, ROC curve analysis showed that both PROMIS-10 physical health and EQ-5D tools were equally able to identify patients who achieved clinically meaningful joint-specific changes of function.

The primary strength of this study was the use of a large countrywide multicentre study cohort with the collection of data at many times postoperatively, allowing a detailed evaluation of comparative responsiveness of the metrics throughout the recovery period, and suggesting broad generalizability. Limitations include the predominance of early postoperative timepoint data collection, with no evaluation beyond one year postoperatively. One-year timeframes are typically reported in studies of the outcome after arthroplasty, and no statistically significant or clinically meaningful changes were apparent between six and 12 months postoperatively, suggesting that the longitudinal data timepoints were sufficient to capture the period of recovery. Although the response rates declined during follow-up, loss to follow-up was minimal and unlikely to bias the estimates.

Understanding which PROMs are most responsive in clinical practice will ensure the collection of high-quality information that best reflects patient-centred health improvements and clinical management. The PROMIS-10 Global Health tool offers superior responsiveness to change compared with the EQ5D in TKA, suggesting that it is a useful tool in this setting. A significant advantage of the EQ-5D compared with the PROMIS tool, however, is the ability to calculate quality-adjusted life years, which can be used to perform health economic analysis. The tools offer a similar evaluation of the quality of life, and it is unlikely that both would be routinely asked of the same patients. Thus, the marginal gain in responsiveness of the PROMIS-10 is unlikely to offer enough benefit to justify replacing the well-entrenched EQ-5D in United Kingdom arthroplasty studies.

**Take home message**

- Both the Patient-Reported Outcomes Measurement Information System 10-Question Short-Form (PROMIS-10) and EuroQol five-dimension score (EQ-5D) tools are sensitive to clinically meaningful change following total knee arthroplasty in cohorts in the United Kingdom, suggesting either is appropriate to evaluate health-related quality-of-life outcomes in clinical studies.
